# The efficacy of Bioelectrical Impedance Analysis (BIA) in monitoring body composition changes during treatment of restrictive eating disorder patients

**DOI:** 10.1186/s40337-014-0034-y

**Published:** 2014-12-04

**Authors:** Charles F Saladino

**Affiliations:** Department of Chemistry and Biochemistry, Misericordia University, Dallas, PA 18612 USA

**Keywords:** Bioelectrical Impedance Analysis (BIA), Anorexia, Bulimia, Body composition

## Abstract

**Reviews:**

Treating restrictive eating disorder patients is metabolically and psychologically complex. Determining body composition is an important diagnostic and treatment option for these patients, because it ascertains whether the acquisition of body mass during refeeding is metabolically appropriate - ideally an approximate 20/80% - 25/75% fat/lean body mass ratio. The purpose of this paper is to evaluate the efficacy of Bioelectrical Impedance Analysis (BIA) during the treatment period of patients with restrictive eating disorders. The search engines PubMed, Medline, and MSN were utilized using combinations of key words, “Bioimpedance Analysis”, “body composition determination”, “eating disorders”, and “anorexia”.

**Conclusions:**

The literature indicates that the use of Bioelectrical Impedance Analysis (BIA) in eating disorder patients to be efficacious in determining body composition during the treatment period, and that only assessing weight changes does not necessarily reflect specific changes in various body compartments. Also, utilizing BIA has the advantage of using each patient as his/her own “control”, potentially allowing for a more individualized nutrition regimen according to the body composition changes observed during treatment.

## Reviews

In the United States, where over 2 trillion dollars yearly is spent on health care, approximately 24 million people of all ages suffer from some form of eating disorder, which collectively forms the highest mortality rate of any mental illness; and the mortality rate associated with anorexia nervosa is 12 times higher than the death rate associated with all causes of death for females 15–24 years old. In addition, almost 50% of people with eating disorders meet the criteria for depression, and only 35% of those receiving any treatment are treated at a facility that specializes in such afflictions [1]. Clearly, treatments for eating disorder patients must include as a goal appropriate restoration of the patients’ overall mental and physical health, including the acquisition of proper body composition.

This paper evaluates the efficacy of utilizing BIA as a potential technique to evaluate body composition during the weight gain process in restrictive-eating disorder patients undergoing treatment. In view of the fact that improper dietary intake can have profound metabolic effects [2–4], using BIA during patient treatment can alert the clinician as to whether or not their treatment diet is appropriate.

The literature on determining body composition is extensive, and various other methods (such as anthropometric measurements and dual x-ray absorptiometry - DXA) are available for this purpose. The following discussion will include comparisons as to the efficacy of BIA vs. some of the other methodologies; but this review focuses on BIA to determine body composition in patients being treated for restrictive eating disorders, such as anorexia nervosa and bulimia nervosa.

For this literature review, three search engines were utilized: PubMed, Medline, and MSN. An assessment of a cross-section of the literature was conducted using combinations of key words that included “Bioimpedance Analysis,” “body composition determination”, “eating disorders”, and “anorexia”. Among the three search engines, in which there was paper overlap, approximately fifty articles were evaluated for relevance to the subject, with 34 chosen to review the use of BIA for measuring body composition - whether or not it applied to patients with restrictive eating disorders.

BIA is an inexpensive and relatively simple method for measuring body composition, first being applied by Hoffer et al. [5] in 1969 to measure total body water and then by Luskaski et al. [6] to determine nutritional status. The instrument uses electrodes to send a harmless very-low level of electric current through the body. Whereas fatty tissue is low in water content and does not conduct electricity well, lean body mass (muscle tissue) is more than 70% water and does conduct an electric current relatively efficiently. BIA measures body fat (FM) (which optimally ranges up to about 25% in women and 20% in men), as well as lean body mass (body cell mass –BCM - plus extracellular mass - ECM) [7,8]. Lean body mass is also referred to as fat free mass (FFM). In addition, the instrument also measures body mass index (BMI) and intracellular and extracellular water.

It is widely accepted that changes in weight and body composition occur with age, health status, and physiological function. In general, aging is associated with a loss of lean body mass and strength; and the elderly can sometimes experience replacement of some muscle mass with fat, but with a stable body weight [9,10], even though weight gain in the young adult into the mid-life years shows an increase in both fat and muscle mass. In addition, intra-abdominal visceral fat and waist circumference often increase with age at a faster rate than total body weight [11–13]; and BMI does not take fat distribution into account [14,15].

Age-related changes in body composition have important correlations to health status later in life. BIA data could theoretically serve as a guideline for good health; and the technique can be used to assess actual body composition and thus overall metabolic well-being. Body composition can correlate to a continuum of health - from longevity and a high level of function on the one hand to morbidity and mortality on the other. Thus, just as early detection of body composition changes resulting from certain diseases allows for early intervention treatments, the restrictive-eating disorder patient could benefit from the clinician having knowledge as to body composition alterations prior to and during therapy.

Mika et al. [16] utilized BIA technology, to evaluated 21 female adolescents with anorexia nervosa (AN) with an initial BMI of 15.5 (15.5 ± 1.1 kg/m^2^) and 19 normal-weight, age-matched female controls, each four times between week 3–15 of refeeding with a hyperenergetic diet. They concluded that changes they observed in extracellular mass (ECM)/body cell mass (BCM) index were due to an increase in BCM, and that multi-frequency phase-sensitive BIA would be a promising tool for assessment of nutritional status and body composition in AN patients.

Mattar et al. [17] compared BIA and Dual x-ray absorptiometry (DXA) to measure FFM and FM in 50 female, underweight patients with anorexia nervosa. For the BIA modality, five equations (Sun, Kushner, Sun, Deurenberg, and Roubenoff - validated in healthy populations) were used for the calculations. Comparisons between BIA and DXA used the sum of the squares of differences and Bland-Altman plots. It was concluded that the best estimates of FFM and FM in the anorexic study group was when the Deurenberg equation was used with BIA, because it took into account height, weight, and age, and it was applicable in adults and adolescents ages 13.4 up to 36.9 and for BMI values of 12.8 - 21.

In order to consider being able to obtain body composition at the bedside, Ghosh et al. [18] evaluated an inexpensive hand-held BIA instrument to measure lean body mass, as compared against DXA in potentially malnourished patients. In this study, body composition analysis was obtained from 17 patients with eating disorders, 7 with chronic alcoholic pancreatitis, and 18 with inflammatory bowel disease The results indicated that in thin and non-obese adults, an accurate two compartment (lean body mass and fat mass) measurement could be made in ten minutes using this hand-held BIA instrument.

In a study utilizing BIA, anthropometry, and DXA to assess body composition, plus a basal endocrine profile was obtained, Bruni et al. [19] sought to identify diagnostic criteria that could distinguish 59 patients with functional hypothalamic amenorrhea mostly related to minimal energy deficiency vs. 59 where a failure in adaptive response to stress was prevalent Subjects with eating disorders had a lower BMI and fat mass (measured with both techniques). Leptin levels were positively associated with fat mass and also with body cell mass indexed to height and BIA phase angle parameters. (This parameter is an indicator of the active lean body compartment and cellular health, and will be further explained below). This study demonstrated the value of BIA, which was corroborated with DXA. Further, in a separate report using the same subjects, Bruni et al. [19] demonstrated that a multivariate analysis model confirmed the utility of integrating endocrine data with the study of body composition; and they reported that BIA proved to be a useful clinical alternative to DXA, especially when considering body cell mass and phase angle.

The parameter of phase angle in BIA is based on total body reactance and total body resistance, independent of weight, height, body fat. Lower phase angles appear to be consistent with either cell membrane degeneration or cell death, whereas higher phase angles are consistent with large quantities of healthy cell membranes and body cell mass. This is because phase angle is positively correlated with capacitance and negatively associated with resistance [20].

Of course, the use of phase angle is not limited to anorexic patients, as seen in a report by Schwenk et al. [21] who found that low phase angle remains an independent adverse prognostic marker of clinical progression and survival in HIV-AIDS patients provided with an antiretroviral therapy that was designed to reduce the risk of wasting. Similarly, Gupta et al. [22] evaluated a case series of 52 patients with histologically-confirmed stage IV colorectal cancer. BIA was conducted on all patients and their respective phase angles were calculated. It was concluded that phase angle would be a prognostic indicator in patients with advanced colorectal cancer. Thus, the phase angle parameter of BIA can be an indicator of overall patient health.

In further considering the efficacy of BIA compared to other modalities of body composition analysis, a study by Beshyah [23] compared fat-free mass (FFM) and% body fat mass (BFM) values derived from total body potassium (TBK), BIA, and DXA in hypopituitary adults before and after a six month treatment with growth hormone (GH). The authors reported that before the GH treatment, there was a strong correlation between the three evaluation modalities, and by the end of the study, they concluded that changes in FFM and BFM values derived by DXA in patients receiving growth hormone vs. placebo were not significantly different from those derived by using TBK or BIA.

A recent study by Krachler et el. [24] was designed to determine whether categories of obesity based upon anthropometric-based estimate of the% fat mass (FM%) had a similar discriminative ability for markers of cardiovascular risk evaluated for FM% by DXA or BIA. The study included 205 males and 388 females ranging in age from 40 – 79 years.

Risk factors including weight, blood pressure, triaglycerols, HDL, cholesterol and fasting glucose were also ascertained. The results showed that for grade 1 hypertension, dyslipidemia and impaired fasting glucose and obesity (defined by FM% and BMI equation) had a higher discriminative power than DXA. However, for grade 2 hypertension, FM% discriminated 1.2% lower (p = 0.05) than DXA and 2.8% (p < 0.01) lower than BIA. They concluded that receiver operation characteristics confirmed BIA as the best predictor of grade 2 hypertension and FM% for grade 1 hypertension.

Moon et el. [25] compared single estimations of fat-free mass (FFM) and tracked FFM in 34 men and 38 women in a control vs. exercise group assessing body composition using BIA equations, DXA, and a 4-C model during weeks 1, 12, and 24 of the study using BIA and DXA compared to a four compartment (4-C) model. Single estimates for DXA and BIA produced high r values (0.79 – 0.95) and low standard errors. Both BIA and DXA equations showed the same significance when comparing groups and times with the 4-C model. Further, individual accuracy for tracking changes were again similar when comparing the two methodologies with the 4-C model in the evaluation of FFM and suggest that the two modalities could be used interchangeably.

Pathophysiological conditions can confound estimates of body composition. For example, Kang et el. [26] conducted a study wherein changes in the difference between BIA and DXA body composition determinations according to edema became an important parameter in peritoneal dialysis patients (PD) patients. After reviewing the data from 409 patients whose body composition was measured by both techniques one or more times, the measurements were divided into four quartiles on the basis of edema index. Significant correlations and intraclass correlations were found for lean body mass (LM), fat mass (FM), and mineral bone content. The authors concluded from the data that when LM is measured by DXA, it is overestimated in PD patients with edema, compared to measurements derived from BIA. However, FM and bone mineral density measured by BIA are overestimated in PD patients, compared to measurements with DXA, especially in patients afflicted with worsening edema; and that the difference between the two modalities grows as the edema worsens. Thus, the authors conclude that a combination of the two methods will allow clinicians to ascertain more accurate body composition evaluations for PD patients with edema.

An interesting study was conducted by Vaz et al. [27] where body composition was analyzed in a group of patients fulfilling DSM-IV criteria for bulimia nervosa (BN). Thus, 43 patients with prior AN (BN-AN group) and 61 without this history (BN-nonAN) were evaluated for height and weight, abdominal diameter, body circumferences, skinfold thickness, and BIA parameters. The results demonstrated that more than 40% of the bulimic patients with an AN history showed a BMI of <20, as well as lower muscle mass and a higher percent of extracellular water. However, these differences were not evident in the second part of the analysis, when only patients with a normal weight range were compared. This indicated that a large number of AN patients tend to retain some clinical traits of the previous condition, remaining in a “subclinical status”. In other words, they were thinner and appeared to have less of a challenge in maintaining a lower weight more than without having had prior AN. Importantly, however, these differences no longer existed in the patients who had achieved a normal weight. This raised interesting questions regarding the boundaries between BN and AN.

In a report by Piccoli et al. [28], 74 AN women with a BMI =10 - 17.5 were evaluated for the frequency of disagreement between BIA and anthropometric estimates of FM and FFM. The results showed a correlation coefficient of 0.8 - 0.9 between the two methods; but the calculations revealed that there was a progressive disagreement between the two techniques when the BMI was <15 kg/m^2^) The authors concluded that although BIA data could be useful in monitoring the patients during refeeding, neither anthropometric methods nor BIA should be used in patients with a BMI of <15.

However, this conclusion appears to conflict with that of Hannan et al. [29] who pointed out that many methods of measuring body composition are time-consuming and require equipment that is often unavailable; but that BIA is simple and inexpensive and can differentiate between lean and fat tissues. Studying 38 anorexic females with highly variable BMI, the authors concluded from the data that the BIA technique compared favorably with other established methods, even in AN patients with a very low BMI.

A negative conclusion about standard BIA was reported by Haas et al. [30] recently who studied 57 AN women with BIA and Bioimpedance vector analysis (BIVA). The authors suggested that because 47% of the patients showed “implausible” results, BIA had “little utility” in these patients, whereas BIVA could be an alternative modality reflecting ECW volume changes and later real tissue mass increases.

Although many of the various body composition studies that have thus far been presented here focus on anorexia, an interesting and recent report by Marra et al. [31] presented data regarding body composition in underweight ballet dancers and constitutionally lean females. The study utilized phase angle measurements to ascertain whether this parameter differed regarding the type of underweight female adolescents and younger women. Thirty AN patients, 10 constitutionally lean individuals, and 15 classic dancers were evaluated by skin fold thickness and BIA. The results indicated that the BIA-derived phase angle discriminated between different forms of underweight, being an effective marker to detect qualitative changes in body composition. Also, in an earlier study by Marra et al. [32], which evaluated the relationship between BMR and BIA in 86 female anorexic patients, multiple regression analysis indicated that phase angle was a predictor of BMR both when solely BIA variables were considered and when combined with either weight and age or fat-free mass.

In a study by Saladino and Dieffenbach [data unpublished, The Use of Bioelectrical Impedance to Monitor Metabolic/Body Composition Changes Resulting from Dietary Treatment in Restrictive Eating Disorder Patients], which assessed 79 restrictive eating disorder patients within an impatient setting and receiving a modified Mediterranean-style diet, it was observed that 37% gained body cell mass interpreted from BIA as lean body mass, and an additional 39% gained lean body mass and fat mass from this diet (p ≤0.05). Importantly, the BIA data showed that the fat mass acquired by this second group of patients still allowed them to achieve a lean body mass to fat ratio of 80/20 - 75/25. The remaining study group lost lean body mass.

It is well-recognized that the issues which lay at the heart of restrictive eating disorder patients are complex. This is exemplified by the work of Van Wymbeleke et al. [33] who studied 87 female AN patients and included BIA to assess body composition. During the treatment period, it was observed that by day eight, resting energy expenditure (REE) increased significantly (13.4%, p <0.01), based upon an increase in fat-free mass, and that the ratio of REE/FFM remained high thereafter.

However by multivariate analysis, they concluded that the rise in this ratio observed during refeeding was significantly related to energy intake, anxiety, abdominal pain, and depressive mood; and they also noted a significant rise in the REE/FFM ratio with physical activity and cigarette smoking. This rise in REE leveled off after recovery from AN. Thus, many variables can affect the results and interpretation of even efficacious BIA data, and that analysis of such data must be careful, thorough, and with as much knowledge about the patient as possible.

## Conclusions

The studies presented in this review demonstrate the metabolic and psychological complexity of understanding and treating restrictive eating disorder patients. However, assessing body mass composition in these patients would be an important component of diagnosis and treatment options; and it is suggested here that such an evaluation would ascertain whether or not the acquisition of body mass during refeeding is metabolically appropriate - ideally achieving an approximate 20/80% - 25/75% fat/lean body mass ratio. In addition, and based upon sometimes conflicting science, it is reasonable to suggest that utilizing BIA techniques to achieve this assessment could be advantageous in patients with eating disorders, such as anorexia nervosa and bulimia nervosa. In addition to the results of the varied studies reviewed here, assessing body composition is also important, because weight changes do not necessarily reflect specific changes in body compartments (including fat free mass and fat mass).

Of course, there are always eating disorder patients with co-morbid disorders that can confound data obtained for a research study or a treatment regimen. However, by utilizing a technique like BIA, clinicians and researchers would have the advantage of using each patient as their own “control” which could potentially allow for a more effective, individualized nutrition regimen according to the body composition changes observed during their treatment period. In addition, BIA might provide information on BMR in anorexic patients and should be explored in evaluating other forms of protein malnutrition. Therefore, in the hands of a qualified clinician who understands the instrumentation as well as human metabolism, it appears that BIA could be a very useful modality in the treatment of patients afflicted with restrictive eating disorders.

## Abbreviations

BIA: Bioimpedance analysis
DXA: Dual-energy X-ray absorptiometry
AN: Anorexia nervosa
BN: Bulimia nervosa
BMI: Body mass index
ECM: Extracellular mass
BCM: Body cell mass
FFM: Fat-free mass
FM: Fat mass
ECW: Extracellular water
GH: Growth hormone
PD: Peritoneal dialysis
REE: Resting energy expenditure
